# Auricular Acupuncture for Exam Anxiety in Medical Students—A Randomized Crossover Investigation

**DOI:** 10.1371/journal.pone.0168338

**Published:** 2016-12-29

**Authors:** Catharina Klausenitz, Henriette Hacker, Thomas Hesse, Thomas Kohlmann, Karlhans Endlich, Klaus Hahnenkamp, Taras Usichenko

**Affiliations:** 1 Department of Anesthesiology, University Medicine of Greifswald, Greifswald, Germany; 2 Institute of Diagnostic Radiology and Neuroradiology, University Medicine of Greifswald, Greifswald, Germany; 3 Institute of Community Medicine, University Medicine of Greifswald, Greifswald, Germany; 4 Institute of Anatomy, University Medicine of Greifswald, Greifswald, Germany; 5 Department of Anesthesia, McMaster University, Hamilton, Canada; National Natural Science Foundation of China, CHINA

## Abstract

Auricular acupuncture (AA) is effective in the treatment of preoperative anxiety. The aim was to investigate whether AA can reduce exam anxiety as compared to placebo and no intervention. Forty-four medical students were randomized to receive AA, placebo, or no intervention in a crossover manner and subsequently completed three comparable oral anatomy exams with an interval of 1 month between the exams/interventions. AA was applied using indwelling fixed needles bilaterally at points MA-IC1, MA-TF1, MA-SC, MA-AT1 and MA-TG one day prior to each exam. Placebo needles were used as control. Levels of anxiety were measured using a visual analogue scale before and after each intervention as well as before each exam. Additional measures included the State-Trait-Anxiety Inventory, duration of sleep at night, blood pressure, heart rate and the extent of participant blinding. All included participants finished the study. Anxiety levels were reduced after AA and placebo intervention compared to baseline and the no intervention condition (p < 0.003). AA was better at reducing anxiety than placebo in the evening before the exam (p = 0.018). Participants were able to distinguish between AA and placebo intervention. Both AA and placebo interventions reduced exam anxiety in medical students. The superiority of AA over placebo may be due to insufficient blinding of participants.

## Introduction

Exam (or test) anxiety is a type of situational anxiety and is reported to be a common problem among university students [1,2]. Exam anxiety often leads to undesirable physiological and mental symptoms and may negatively influence academic performance [2,3]. Various mindfulness-based behavioral interventions have been shown to be effective in reducing exam anxiety and stress among university students [4,5]. Some of these methods (e.g. expressive writing) have even been claimed to improve academic performance [6]. However, all of these methods are time consuming, which makes the routine use of cognitive and behavioural interventions in the treatment of anxiety immediately before an upcoming exam difficult [4,5].

Auricular acupuncture (AA) is a complementary medicine technique, which is physiologically based on the mechanical stimulation of cranial nerves [7]. AA has already successfully been used to treat situational anxiety in clinical settings, such as dental and preoperative anxiety [8–10]. For exam anxiety, AA was studied in only one prospective observational study in medical students without a control group [11]. Since this study did not provide sufficient information for further research, we tested the methodology of the AA intervention as well as the outcome assessment in treatment of pre-exam anxiety using a preliminary pilot investigation [12]. This pilot investigation informed the final study design and provided data to calculate the sample size for a subsequent randomized controlled study.

The aim of the present study was to investigate whether AA can reduce exam anxiety in medical students in comparison with placebo and no intervention conditions in a randomized crossover investigation.

## Methods

### Study design and randomization

This prospective randomized, placebo controlled, single blinded crossover trial was performed between April and July 2012 at the University of Greifswald, Germany. The participants were recruited via announcement in March 2012 before the first anatomy exam in April according to the following eligibility criteria: undergraduate medical students in their first year of study with no fundamental knowledge about and experiences with acupuncture, undergoing three oral anatomy exams within one month, without any history of alcohol abuse or use of opioid or psychotropic medication and with an American Society of Anesthesiologists physical status score of I-II. None of the students were taking any medications or recreational drugs at the time of the study and all of them were paid for their participation. The follow-up was finished on the day of the last anatomy exam in July 2012.

**Fig 1:**
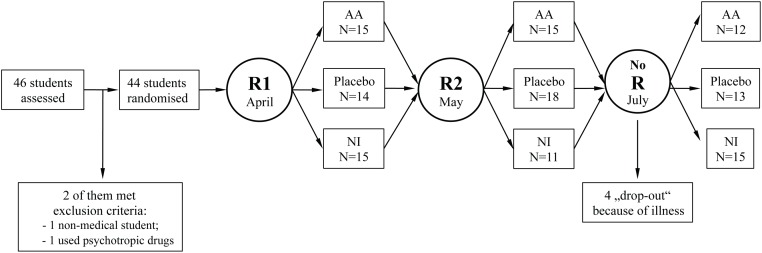
Flow of the study. First randomization (R1) was performed by drawing wrapped pieces of paper with hidden numbers ranging from 1 to 3 before the first exam. Second randomization (R2) was performed before the second anatomy exam by flipping a coin; no further randomization was necessary before the last exam in July. R: randomization; AA: auricular acupuncture; NI: no intervention.

The research project was approved by the Institutional Ethics Committee of the University Medicine of Greifswald (reference no. BB 49/12). The trial was registered at clinicaltrials.gov (registration number NCT02920164) after the enrollment of the participants was started since initially the authors regarded the project as an experimental investigation. The authors confirm that all ongoing and related trials for this drug/intervention are registered. The written informed consent was obtained from each participant after the nature of the study procedures was explained. As all students took three comparable anatomy exams with an interval of one month, each of them was randomly assigned to the AA, placebo or no intervention condition at the evening prior to a scheduled exam by drawing slips of paper with the numbers 1, 2 or 3 out of a hat. Each number corresponded to an intervention, as determined a priori: 1 = AA, 2 = placebo, 3 = no intervention before the first exam (Fig 1).

Before the second exam, participants were randomly assigned to one of the two remaining conditions by flipping a coin. The investigator, who performed the randomization, ensured that the participants could not have been randomized again to the condition they had before. Before the last exam, no further randomization was necessary. The investigator informed the acupuncturist about the assignment of the next participant immediately after the randomization procedure and prior to any intervention.

### Study interventions

A licensed acupuncturist with more than five years of experience with this technique applied AA at the acupuncture points MA-IC1 (Lung), MA-TF1 (ear Shenmen), MA-SC (Kidney), MA-AT1 (Subcortex) and MA-TG (Adrenal gland) bilaterally according to the methodology, which was previously described in detail elsewhere [12]. Indwelling fixed 'New Pyonex' needles (length 1.5mm, diameter 0.22mm; Seirin Corp, Shizuoka City, Japan) embedded in a skin-colored adhesive tape were used for AA. The participants were instructed by the acupuncturist to stimulate the auricular needles for 3–5 minutes, if they felt anxious.

**Fig 2:**
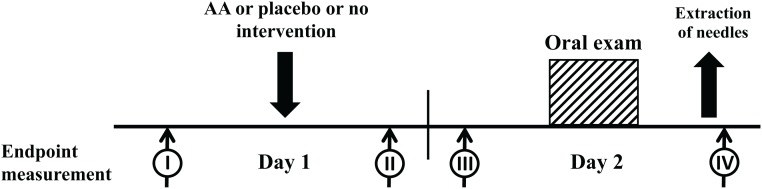
Timeline of the investigation with endpoint measurements. Time I: baseline; time II: evening of the day before exam; time 3: immediately before the anatomy exam; time IV: after exam. Auricular acupuncture (AA) was performed in the evening before the day of exam (time I) using indwelling fixed needles, which remained in situ and were removed after the exam (time IV). Exam anxiety was measured using the German version of Spielberger’s State-Trait-Anxiety Inventory (STAI) and 100 mm visual analogue scale (VAS-100) at times I, II and III, as was heart rate and blood pressure. Duration and quality of sleep (over the course of the preceding 1 night, 1 week and 6 months) were enquired about at time III. Immediately after the exam, at time point IV, exam performance (passed or failed) and the quality of participants’ blinding were recorded.

For the placebo procedure, 'New Pyonex' placebo needles were attached to five sites on the helix of the auricle bilaterally. 'New Pyonex' placebo needles have the same appearance as AA needles but consist of self-adhesive tape only [13]. In order to avoid potential physiologic effects of acupressure, the participants were not instructed to stimulate the attached 'New Pyonex' placebo needles. AA and placebo needles were left in situ until the next day and were removed out of sight of the participants after the exam by the investigator, who was not involved in acupuncture procedure (Fig 2).

If the participants were assigned to the no intervention condition, they remained seated in the examination room for 10 to 15 min, which is the same amount of time an application of the needles would have taken. During that time, the investigator conducted a conversation with the participants about leisure activities, place of birth, and opinions on the study facility, thereby, avoiding the topic of the upcoming exam.

For blinding purposes, participants were told that the study’s aim was to investigate two different combinations of AA points as treatment methods for pre-exam anxiety in comparison with no intervention. Participants had no knowledge which condition they had been randomized to, except for the no intervention condition.

### Outcome measures

Pre-exam anxiety was measured in the evening prior to the exam; before the intervention (Time I); after the intervention (Time II); and immediately before the exam (Time III, Fig 2) using a 100 mm visual analogue scale (VAS-100; from 0 = no anxiety to 100 = maximum imaginable anxiety) as primary outcome. Additionally state and trait anxiety levels assessed with the German version of Spielberger’s State-Trait-Anxiety Inventory (STAI; ranging from 20 = no anxiety to 80 = maximum imaginable anxiety; [14]) were registered at all three time points. In the morning of the exam the participants were asked to fill out a questionnaire about the quality (6-point-scale ranging from 1 = excellent sleep to 6 = no sleep at all) and duration of sleep the night before as well as the duration of sleep in the preceding i) week and ii) in the previous six months. Blood pressure and heart rate were measured before and after each intervention as well as before and after each exam (Time I-IV, Fig 2). Immediately after the exam, at Time IV, exam performance (passed or failed) and the quality of participant blinding were recorded.

### Statistical analysis

The sample size was calculated based on a prospective pilot study [12] by determining the two-sided level of significance at 0.015 (three-period crossover investigation) and power at 85% for a paired sample *t*-test. Expecting to find a 25% difference in anxiety level between the different study conditions and using the mean and standard deviation values measured in the pilot investigation using STAI State-anxiety, the number of participants needed was calculated to be 43. Taking into account potential drop-out/withdrawal cases, the sample size was inflated to a total of 46 volunteers.

Baseline characteristics as well as the differences between the study conditions at different time points were analyzed using paired sample *t*-tests, Holm-Bonferroni adjusted for multiple comparisons. Fisher’s exact test was used to analyze the success of volunteer blinding. Data analysis was performed using IBM SPSS Statistics Software for Mac (Version 19.0.0, IBM Corp., New York, USA). All data are presented as mean (standard deviation) unless otherwise stated, two-sided Holm-Bonferroni-adjusted *P*-values < 0.05 were regarded as significant.

## Results

46 students agreed to participate; two of them did not fulfill the inclusion criteria (Fig 1). 44 students (all Caucasian, 35 females) aged 23 (3) were enrolled in the study. Four female participants missed the third session because of illness (Fig 1), their data were treated as missed data.

**Fig 3:**
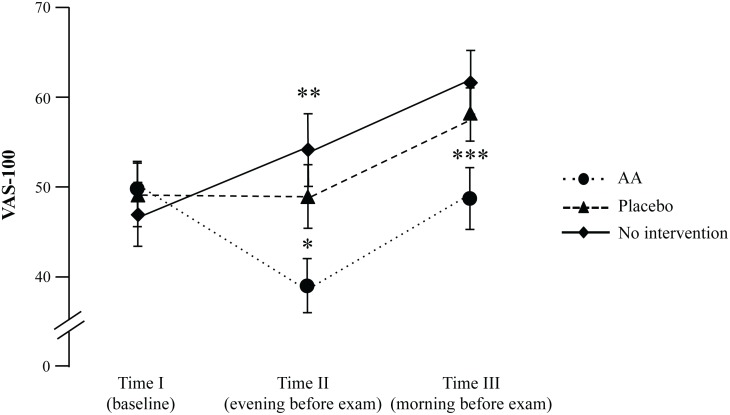
Exam anxiety measured using Visual Analogue Scale 100mm. Time I: baseline; time II: evening of the day before exam; time 3: immediately before the anatomy exam. * Holm-Bonferroni adjusted *P* = 0.018 for AA vs. placebo; ** Holm-Bonferroni adjusted *P* = 0.003 for AA vs. no intervention at time II and *** *P* < 0.003 for AA vs. no intervention at time III. Data given as mean (standard error of mean).

The baseline anxiety levels (Time I) were comparable among all three trial conditions. Anxiety levels measured with VAS 5 hours (2) after an intervention in the evening prior to the anatomy exam (Time II) decreased after AA in comparison with baseline values at Time I (mean difference (MD) = 10.5; 95% CI 5.3, 15.8; t_40_ = 4.0, *P* < 0.001, d = 0.6, 95% CI 0.3, 1.0; Table 1, Fig 3).

**Table 1:** Outcome measures of the investigation presented as mean (SD).

Parameter	Time of measurement	Intervention		
		AA	Placebo	No intervention
**Exam anxiety (VAS-100 mm)**	I (baseline)	50 (21)	49 (23)	47 (24)
	II (after intervention)	**39 (20)**	49 (24)*	54 (25)**
	III (before exam)	**49 (22)**	58 (21)*	62 (23)**
**Trait anxiety**	I	44 (11)	45 (12)	43 (11)
**State anxiety**	I	55 (11)	53 (12)	54 (11)
	II	**47 (11)**	52 (12)*	57 (13)**
	III	**53 (11)**	54 (10)*	60 (11)**
**Duration of sleep (h)**	Preceding 6 months	7.7 (1)	7.7 (1)	7.7 (1)
	Preceding week	6.9 (1)	7.0 (1)	7.0 (1)
	Night before exam	7.0 (2)	7.3 (1)	6.8 (2)
**Passed exam, N (%)**	After exam	34 (81)	32 (79)	33 (84)

Statistically significant differences between 3 study conditions (in bold letters) revealed with paired sample *t*-tests with Holm-Bonferroni-adjustment for multiple comparisons. VAS-100: Visual Analogue Scale 100 mm.

* *P* ≤ 0.05 for comparisons of auricular acupuncture (AA) vs. placebo

** *P* < 0.01 for comparisons of AA vs. no intervention.

VAS-100 anxiety level at Time II was lower than after no intervention (MD = 13.4; 95% CI 5.6, 21.1; *t*_32_ = 3.5; *P* = 0.003, d = 0.6) as well as lower after AA than after placebo (MD = 10.2; 95% CI 2.7, 17.7; *t*_40_ = 2.7; *P* = 0.018, d = 0.4). On the morning of the exam (Time III), VAS-100 anxiety level after AA was also lower than after no intervention (MD = 12.3; 95% CI 5.8,18.8; *t*_36_ = 3.9; *P* < 0.003, d = 0.6) and in comparison with placebo (MD = 9.65; 95% CI 1.3,18.0; t_39_ = 2.3; *P* = 0.05; d = 0.4).

In line with the findings of the primary outcome, state anxiety assessed with STAI at Time II was also reduced after AA in comparison with placebo (MD = 4.4; 95% CI 0.7, 8.1; t_40_ = 2.4; *P* = 0.021, d = 0.4) and as compared to no intervention (MD = 9.9; 95% CI 6.0,13.9; *t*_33_ = 5.1; *P* = 0.003; d = 0.9). State Anxiety after placebo at Time II was also lowered if compared to no intervention (MD = 5.2; 95% CI 1.2, 9.2; *t*_36_ = 2.6; *P* = 0.024, d = 0.4). At Time III, state anxiety after AA (MD = 6.7; 95% CI 3.5,9.9; *t*_37_ = 4.2; *P* = 0.003, d = 0.7) and after placebo (MD = 5.1; 95% CI 1.9,8.2; *t*_40_ = 3.3; *P* = 0.004, d = 0.5) was reduced as compared to no intervention. The trait anxiety, assessed with STAI was 44 (11), which significantly exceeds the mean found in the norm sample for the female population aged between 15 and 29 years (36 (10); t_35_ = 4.1; p < 0.001, d = 0.7; 14, Table 1).

The duration and quality of sleep, blood pressure, heart rate and exam performance were comparable among the three study conditions (Table 1 and S1 Table). Being asked after the exam about their opinion on allocation to the study condition, the participants could distinguish between AA and placebo intervention: for the AA condition, 34 participants thought that they had received acupuncture vs. 7 for the placebo condition (*P* < 0.001; Table 2).

**Table 2:** Participants’ opinion about the allocation to study condition/intervention.

	Intervention	
	Auricular Acupuncture	Placebo
**It was verum**	34 (77)	7 (15)
**It was placebo**	6 (14)	21 (48)
**Do not know**	1 (2)	8 (18)

Data is presented as number of participants (%).

## Conclusion

This randomized crossover trial demonstrated that both auricular acupuncture (AA) and placebo reduced exam anxiety in comparison with no intervention in medical students, whereas AA yielded stronger effects than placebo procedure.

Without any intervention, the level of anxiety, measured with both STAI and VAS-100, increased constantly before the upcoming exam (Fig 3). These results are in line with the findings of Brockmeyer et al. [15]. Exam anxiety decreased by up to 20% from baseline after AA in comparison to placebo procedure and no intervention. The largest effect size of AA over placebo procedure and no intervention was measured using VAS-100 in the evening after the intervention on the day before the exam. The effect size observed in the present study is comparable to the findings of our pilot investigation [12] and other previous studies of AA and situational anxiety [8–11]. For example, Karst et al. [9] reported that state anxiety scores decreased by about 18% from baseline to after AA treatment of dental anxiety in 19 patients. Likewise, Michalek-Sauberer et al [10] demonstrated a reduction in STAI state anxiety levels by about 15% from baseline to after AA in 61 patients for dental procedures. However, both investigations failed to find the difference between verum AA and placebo procedures.

In our investigation, placebo procedure was found to reduce exam anxiety in comparison to no intervention in medical students as well, although this effect was not as strong as the reduction of pre-exam anxiety through AA. Even if the majority of participants could distinguish between verum and placebo procedure, we are hesitant to say that the difference between AA and placebo occurred due to a bias of potential “unblinding” because of two aspects: i) due to “unblinding”, the placebo effect should have disappeared, however there was sustained effect of placebo over the no intervention condition, as measured by both VAS-100 and STAI (Table 1, Fig 3); ii) questioning about the opinion on the allocation to the study condition took place at the end of the study after the exam, meaning that the exact time of potential “unblinding” is unclear. We could not determine the time of “unblinding” in this study and this fact remains the main limitation of our investigation. Moreover, since the participants were instructed to stimulate the needles in case they start to feel anxious only before the AA condition, this action could not be controlled in the placebo condition and may have diminished the difference between AA and placebo due to the weak physiological effect of acupressure applied to 'New Pyonex' placebo needles [16]. Furthermore, on the day before the exam, 3 participants did not document their anxiety levels. They reported to have forgotten about it because of their high stress levels immediately before the exam.

As expected, STAI trait anxiety scores did not change during the course of the investigation. This is unsurprising as they are thought to reflect a stable personal characteristic that remains constant over time and between events [14]. The mean value of trait anxiety in the study group significantly exceeded the mean found in the general female population aged between 15 and 29 years [14]. This is in agreement with previous findings about increased anxiety levels and lower self-confidence among female medical students [17] and explains the “natural” selection of predominantly anxious females that volunteered to participate in our study.

Despite the expectations based on previous results [6, 8–12], we could not observe the beneficial effect of AA or placebo on quality and duration of sleep as well as exam performance in participants of our study. The stability of hemodynamic parameters and the absence of side effects and complications confirm the respective findings of previous investigations [6, 8–13].

This trial followed the CONSORT guidelines for specific requirements of acupuncture studies [18,19]. The randomized crossover design and the use of a formulaic auricular acupuncture (constant pattern of cranial nerves stimulation) rather than individualized acupuncture have minimized potential biases. The dropout rate of 9% was low.

Regarding the above-mentioned limitations of the present investigation we suggest that future studies should examine larger samples to compensate for dropout rates and incomplete data. The “parallel arms” approach might be considered in order to prevent any exchange of participant experiences and intra-individual “carry-over” experience, which may have contributed to the potential “unblinding” in our investigation. Also the number of participants, who have stimulated the needles by pressing, if they felt anxious, should be verified in future investigations, since the stimulation (or not stimulation of the needles) might have caused the effect bias. Furthermore, the measurement of non-invasive stress biomarkers as salivary cortisol, salivary α-amylase or catecholamines in urine might strengthen the findings of any future investigations, giving further insights into the biological mechanisms of AA.

In order to evaluate the clinical significance of the AA effect, this technique should be compared with methods that are commonly used for treatment of exam anxiety, such as relaxation techniques, biofeedback and systematic desensitization [4–6, 20–22]. Moreover, after appropriate investigations, AA might be used to treat pre-operative anxiety in surgical patients, constituting serious alternative for benzodiazepines, commonly used for this purpose in clinical practice [9,10,23].

In conclusion, both auricular acupuncture and placebo procedure were shown to be effective in reducing levels of exam anxiety in medical students. The superiority of verum AA over placebo AA and no intervention is considered to be due to stimulation of cranial nerves, but may have been increased in effect by insufficient participant blinding.

## Supporting Information

S1 Table Blood pressure and heart rate during the study conditions given as mean (SD). (DOCX)

S1 File Ethics commission application. (PDF)

S2 File Pilot investigation. (PDF)

S3 File Study Data. (SAV)

S4 File Consort Checklist. (PDF)
