# Supplementary material for: Auricular Acupuncture for Exam Anxiety in Medical Students—A Randomized Crossover Investigation
Source: PLoS One. 2016 Dec 29;11(12):e0168338. doi: 10.1371/journal.pone.0168338 (PMC5198977; doi:10.1371/journal.pone.0168338)
Supplement: S1 Table — (DOCX) [file pone.0168338.s001.docx]

**S1 Table.** Blood pressure and heart rate during the study conditions given as mean (SD).

| **Parameter** | **Time of measurement** | **Intervention** | | |
| --- | --- | --- | --- | --- |
|  |  | **AA** | **Placebo** | **No intervention** |
| **Systolic blood pressure (mmHg)** | II | 121 (13) | 121 (14) | 123 (15) |
|  | III | 138 (16) | 139 (15) | 141 (19) |
|  | IV | 130 (14) | 131 (16) | 132 (17) |
|  |  |  |  |  |
|  | I | 77 (10) | 79 (10) | 79 (10) |
| **Diastolic blood pressure (mmHg)** | II | 75 (9) | 75 (10) | 74 (9) |
|  | III | 84 (11) | 83 (9) | 83 (11) |
|  | IV | 81 (10) | 82 (10) | 80 (9) |
|  |  |  |  |  |
|  | I | 78 (15) | 75 (12) | 80 (17) |
| **Heart rate (bpm)** | II | 75 (12) | 76 (12) | 78 (14) |
|  | III | 103 (19) | 106 (20) | 110 (22) |
|  | IV | 95 (15) | 102 (14) | 99 (17) |
