# Supplementary material for: Auricular Acupuncture for Exam Anxiety in Medical Students—A Randomized Crossover Investigation
Source: PLoS One. 2016 Dec 29;11(12):e0168338. doi: 10.1371/journal.pone.0168338 (PMC5198977; doi:10.1371/journal.pone.0168338)
Supplement: S1 File — (PDF) [file pone.0168338.s002.pdf]

Titel: Auricular acupuncture (AA) for pre-exam anxiety - a blinded randomized crossover study in healthy volunteers

geplante Dauer 04/2012 – 07/2012

Leiter der Studie PD Dr. T. Usichenko
Klinik für Anästhesiologie und
Intensivmedizin
Universitätsmedizin Greifswald
Fleischmannstraße 42-44
D-17487 Greifswald
Telefon: 049-(03834) 865893
Telefax: 049-(03834) 865802
E-Mail: taras@uni-greifswald.de

Contents

	CONTENTS	2
1.	GENERAL INFORMATION	3
1.1	Institution and investigators	3
1.2	Synopsis	4
2.	SCIENTIFIC BACKGROUND AND AIMS OF THE STUDY	5
2.1	Background	5
2.2	Aims of the study	5
3.	ETHICAL ASPECTS	6
3.1	Benefit-risk considerations	6
3.2	Protection of patients	6
4.	STUDY DESIGN	7
5.	PATIENTS' SELECTION	7
5.1	Inclusion criteria	7
5.2	Exclusion criteria	7
5.3	Discontinuation criteria	7
6.	TREATMENT PLAN	8
6.1	Group allocation	8
6.2	Study procedure	8
6.3	Outcome measures	9
7.	SAMPLE SIZE AND STATISTICS	10
8.	REFERENCES	11
9.	APPENDICES	
	Appendix A (Informed Consent)	12
	Appendix B (Special Participants Information)	13
	Appendix C (Study Protocol)	16

1. GENERAL INFORMATION

1.1. Institutions and Investigators

Institutions: Klinik für Anästhesiologie und Intensivmedizin
Universitätsmedizin Greifswald
Fleischmannstraße 42-44
D-17487 Greifswald
Telefon: 049-(03834) 865893
Telefax: 049-(03834) 865802

Principal Investigator: PD Dr. Taras Usichenko
Klinik für Anästhesiologie und Intensivmedizin
Universitätsmedizin Greifswald

Principal Investigator on site: Catharina Klausenitz
Studentin der Humanmedizin
Universitätsmedizin Greifswald

1.2. Synopsis

Title of the study:	Auricular acupuncture (AA) for pre-exam anxiety - a blinded randomized crossover study on healthy volunteers
Study period:	04 / 2012 – 07 / 2012
Principal Investigator:	PD Dr. T. Usichenko<br>Department of Anesthesiology and Intensive Care Medicine<br>University Medicine of Greifswald
Aim of the study:	To investigate the anxiolytic effect of auricular acupuncture (AA) in comparison to placebo acupuncture and no intervention during oral exams in anatomy at the University of Greifswald
Design:	Prospective blinded randomized crossover trial
Intervention:	1. AA using indwelling fixed needles, retained 24 h *in situ*<br>2. AA using placebo needles
Number of volunteers:	N = 33
Inclusion criteria:	Healthy medical students at the University of Greifswald<br>Participants of the anatomy exams in spring/summer 2012<br>Without previous anxiolytic, sedative and analgesic medication<br>No former experiences with acupuncture<br>No pregnancy or lactating<br>Informed consent
Outcome measures:	Anxiety level<br>Heart rate, blood pressure<br>Salivary cortisol

2. SCIENTIFIC BACKGROUND AND AIMS OF THE STUDY

2.1 Background

2.1.1 Pre-Exam Anxiety

Pre-exam anxiety is widely spread among students and has great impact on their life. It is well known that the physiological parameters, such as blood pressure and heart rate as well as subjective anxiety level, measured using various validated inventories, increase before an exam (1,2,3). Moreover, recent investigations have shown, that high pre-exam anxiety levels can influence the exam scores (3,4).

2.1.2 Auricular acupuncture for pre-exam anxiety

Several interventions have demonstrated to reduce the fear. Writing about testing worries and cognitive behavioral therapy are the most common effective methods described previously (4,5). Concerning auricular acupuncture (AA), it has been shown that this intervention is effective in reducing clinical anxiety, such as pre-surgery (6) and pre-dental treatment anxiety (7). Regarding pre-exam anxiety, there is only one published investigation, which aimed to show the effects of AA for pre-exam anxiety. However, this prospective investigation lacks a control group to compare with (8). Therefore we are going to perform a prospective crossover investigation with three conditions (periods): verum acupuncture, placebo acupuncture and no intervention (standard condition), where each volunteer will serve as a self-control.

2.2 Aims of the Study

Primary objective:
To compare the anxiolytic effect of AA vs. placebo AA and no intervention measured using a validated inventory.

Secondary objectives:
To investigate the influence of AA on blood pressure, heart rate and salivary cortisol as the effects of pre-exam stress reaction under the three conditions during the study.

3. ETHICAL ASPECTS

The present study protocol complies with the requirements of the following directives and guidelines:

- Declaration of Helsinki (48 General Assembly, Somerset West, South Africa, 1996)
- Good Clinical Practice in the Conduct of Clinical Trials on Medicinal Products for Human Use (Brussels, 20.06.96, E6GCPD12/01 May 96)
- Berufsordnung für die deutschen Ärzte (Dtsch Ärzteblatt 94, 2354 - 2363, 1997)
- Standards of the Department of Anesthesiology and Intensive Care Medicine, Ernst Moritz Arndt University of Greifswald

3.1 Benefit-risk consideration

The patients receiving AA are expected to have benefit in reducing the intensity of anxiety; thus the improvement of the exam performance is hypothesized. The prognosis for the beneficial effect of AA is based on the results of randomized controlled studies (6,7).

The most common side effects of AA, described in case reports, were endocarditis and perichondritis (9,10). Therefore, we shall exclude the patients at risk (with damaged or prosthetic cardiac valves) for endocarditis from our investigation. We did not observe serious side-effects of AA in more than 600 patients treated with indwelling fixed needles during the prospective clinical trials on analgesic effects of AA.

3.2 Protection of Participants

Ethics Committee

The protocol including the copies of the “Informed Consent Form” and ”Special Information for Participants” will be submitted to the Ethics Committee of the University of Greifswald. Written approval of the study protocol and the proposed “Informed Consent Form” and “Special Information for Participants” should be obtained before the start of the study.

Participant information

The participants will be informed both orally and in a written form about the study procedure, potential benefits and risks of the participation in the study, the right to withdraw from the study at any time, and to obtain written informed consent prior to any study procedures.

Insurance

The question about participants’ insurance (any harms and injuries, caused by the study or during the study) will be clarified with Allianz Insurance Company.

4. STUDY DESIGN

This will be a prospective blinded randomized crossover study at one center –the University of Greifswald. The total duration of the study will be six days for each volunteer. These 6 days are divided in three parts, two days each, because of three anatomy exams (2 x in spring and 1 x in summer). Two days of investigation are necessary for each exam. The ethics committee should approve the design of the study. A total of 30 healthy volunteers scheduled for the oral exams in anatomy will be recruited according to participants selection criteria.

5. PARTICIPANTS' SELECTION

5.1 Inclusion Criteria

1. Medical students at the University of Greifswald
2. Going to take part in oral exams of human anatomy
3. Participants without previous anxiolytic medication
4. Participants without any experiences with acupuncture
5. Patients who have given written informed consent

5.2 Exclusion Criteria

1. Local auricular skin infection
2. Pregnant or lactating women
3. Participants with prosthetic or damaged cardiac valves, intracardiac and intravascular shunts, hypertrophic cardiomyopathy and mitral valve prolaps (risk of bacterial endocarditis according to guidelines of AHA)
4. Participants who are unable to understand the consent form
5. History of psychiatric disease, alcohol and drug abuse

5.3 Discontinuation Criteria

1. In case of severe intercurrent disease
3. All participants are free to withdraw from participation in this study at any time, for any reason, without penalty or loss of benefits.

6. TREATMENT PLAN

6.1 Group Allocation

Following the signing of the informed consent for participation in this study (**Appendix A**) along with the Special Participants Information for auricular acupuncture for reducing pre-exam anxiety (**Appendix B**), all study participants will be consecutively randomized to receive either verum AA, placebo AA or no intervention on the day before the anatomy exam. Randomization will be performed using a list of random numbers.

6.2 Study procedure

Five ear acupuncture points (according to Nogier international classification 31, 51, 55, 95, 100 will be needled bilaterally in patients from the study group (Figure 1). The choice of the ear acupuncture points is based on previously reported data about anxiolytic effects with AA (5,11). Disposable indwelling steel ear acupuncture needles “New Pyonex” Seirin Corp. (Japan), sized 0.22 x 1.5 mm, will be used for this procedure.

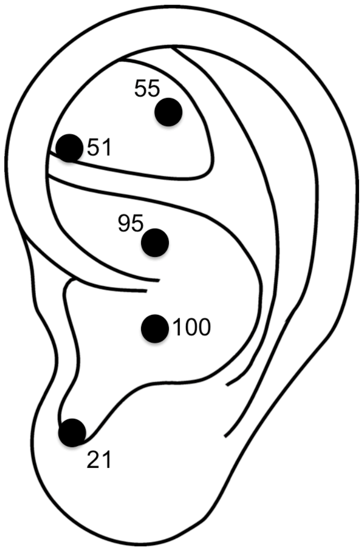

Figure 1. Sites of external auricle, used to treat anxiety

The needles will be placed on the day before the exam, fixed with skin-colored adhesive tape and retained in the ear until the following day after the exam. Before needle insertion, the ears should be completely disinfected with alcohol swabs.

For placebo AA, special placebo needles “New Pyonex Placebo”, manufactured for the purpose of placebo investigation by Seirin Corp., will be used. The placebo AA needles look like the verum needles (same adhesive tape) except for the needles itself, which are absent.

Physiological parameters, such as blood pressure and heart rate, are measured before and after the insertion of the needles as well as before the exam. In addition at the day of the exam there will be a sample of salivary given by each volunteer for measuring the level of cortisol (Figure 2).

Anxiety scores are measured using STAI (State-Trait-Anxiety Inventory) and VAS (Visual Anxiety Scale) which will be filled in by the participants before and after the insertion of the acupuncture needles as well as before the exam. A questionnaire about the duration and quality of sleep will be filled in the morning after the acupuncture treatment.

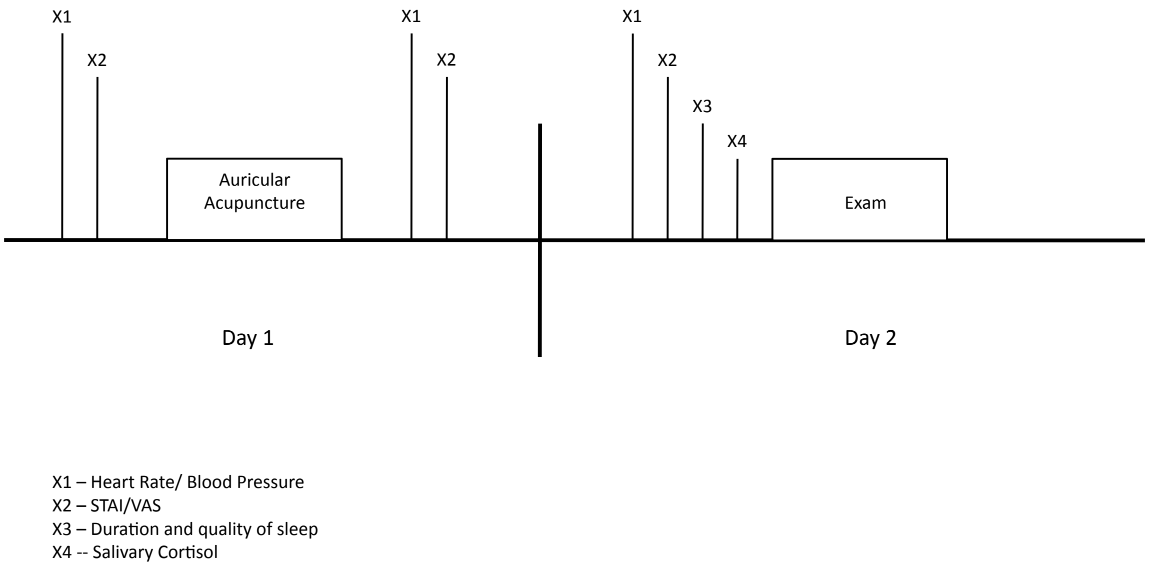

Figure 2. Flow diagram of the study

The needles will be retained *in situ* until the day of the exam. In case of a local inflammation or any adverse effects potentially connected with ear needling, acupuncture needles will be immediately removed and participants will be withdrawn from the study.

6.3 Outcome Measures

Primary outcome measure: level of pre-exam anxiety using Visual Anxiety Scale-100 (where 0=no anxiety at all; 100=maximal anxiety imaginable)

Secondary outcome measures: (see Figure 1)
-State and trait anxiety, using validated STAI (State-Trait-Anxiety Inventory)
-duration and quality of sleep
-exam scores
-physiologic parameters – heart rate (bpm), blood pressure (mmHg), salivary cortisol
-the quality of volunteers' blinding (asking their opinion about the group allocation after the study)

7. SAMPLE SIZE AND STATISTICS

In order to calculate the appropriate sample size we set the level of significance to 0.01 (crossover investigation with multiple comparisons) and power at 85%. Taking the mean and standard deviation values of anxiety level, measured with STAI in our pilot investigation in 11 healthy volunteers and using the online sample size calculator (12) the number of volunteers was calculated to be 27. Taking into account potential drop-out/withdrawal rate of 10-20%, the sample size was set to a total of 33 volunteers. Normally distributed continuous data will be compared using the Student's *t*-test and an analysis of variance for paired observations, as appropriate. Skewed data will be compared using Friedman. Chi-square test will be used to analyze the success of volunteers' blinding and the incidence of side effects. SPSS Statistics Software for Mac (Version 19.0.0) will be used for data analysis.

8. REFERENCES

1. Zhang Z, Su H, Peng Q, Yang Q, Cheng X. Exam anxiety induces significant blood pressure and heart rate increase in college students. Clin Exp Hypertens 2011;33(5):281-6. Epub 2011 Apr 5.
2. Latas M, Pantic M, Obradovic D. Analysis of test-anxiety in medical students. Med Pregl 2010 Nov-Dez;63(11-12):863-6.
3. Neuderth S, Jabs B, Schmidtke A. Strategies for reducing test anxiety and optimizing exam preparation in German university students: a prevention-oriented pilot project of the University of Würzburg. J Neural Transm 2009 Jun;116(6):785-90. Epub 2008 Sep 23.
4. Ramirez G, Beilock SL. Writing About Testing Worries Boosts Exam Performance in the Classroom. Science 2011;331:211-3.
5. Orbach G, Lindsay S, Grey S. A randomised placebo-controlled trial of a self-help Internet-based intervention for test anxiety. Behav Res Ther 2007 Mar;45(3):483-96. Epub 2006 Jun 30.
6. Wang MD, Peloquin S, Kain Z. The Use of Auricular Acupuncture to Reduce Preoperative Anxiety. Anesth Analg 2001;93:1178–80.
7. Karst M, Winterhalter M, Münte S, Francki B, Hondronikus A, Eckhardt A, Hoy L, Buhck H, Bernateck M, Fink M. Auricular Acupuncture for Dental Anxiety: A Randomized Controlled Trial. Anesth Analg 2007;104(2):295-300.
8. Ogal H, Ogal M, Hafer J, Henning J, Brockemeyer H, Krach R, Krumholz W. Auricular Acupuncture for exam anxiety. Dt Ztschr f Akup 2004;47:6-12.
9. Rampes H. Adverse reactions to acupuncture. In: Medical acupuncture-a western scientific approach. Ed: Filshie J, White A. Chirchill livingstone, 1998.
10. Lee McIlwain. Subacute bacterial endocarditis following ear acupuncture. Int J Cardiol 1985;7:62-3.
11. Lewis GBH. An Alternative Aproach to Premedication: Comparing Diazepam with Auriculotherapie and a Relaxation Method. American Journal of Acupuncture 1987;15(3):205-14.
12. http://www.quantitativeskills.com/sisa/calculations/samsize.php (accessed 23.04.2012).

APPENDICES

Appendix A

Untersuchung zu Ohrakupunktur gegen Prüfungsangst bei Medizinstudenten

Name, Vorname des Probanden:

Geburtsdatum:

Ich wurde von dem unterzeichnenden Arzt über Art, Ziel und Ablauf der Studie und die zu erwartenden Wirkungen und Risiken aufgeklärt und habe die spezielle Probandeninformation gelesen und verstanden. Ich hatte auch ausreichend Gelegenheit, Fragen zu stellen und habe momentan keine weiteren Fragen.

Meine Teilnahme an der Studie ist freiwillig und ich kann jederzeit, auch ohne Angabe von Gründen, die Teilnahme an der Studie beenden, ohne dass mir dadurch Nachteile entstehen. Während meiner Teilnahme werde ich mich an die Anordnungen der Ärzte halten.

Ich bin damit einverstanden, dass die im Rahmen der Studie erhobenen Daten zur wissenschaftlichen Auswertung in anonymer Form verwendet werden dürfen. Weiterhin bin ich damit einverstanden, dass meine personengebundenen Daten zur Qualitätskontrolle und Qualitätsüberwachung der klinischen Prüfung durch die interne Qualitätssicherung der Klinik für Anästhesiologie und Intensivmedizin und die zuständige Überwachungsbehörde eingesehen werden dürfen.

Ich erkläre mich bereit, zu den obigen Bedingungen an dieser Prüfung teilzunehmen. Ich unterliege derzeit keiner Sperrfrist durch ein anderes Institut.

Greifswald, den Unterschrift des Probanden:

Greifswald, den Unterschrift des aufklärenden Arztes:

Appendix B

Untersuchung zu Ohrakupunktur gegen Prüfungsangst bei Medizinstudenten

Kurztitel: Ohrakupunktur & Prüfungsangst

Prüfarzt: PD Dr. T.I. Usichenko, Tel.: 86 5893

Doktorand: Catharina Klausenitz

Ort der klinischen Studie: Ernst-Moritz-Arndt-Universität Greifswald
Medizinische Fakultät
Klinik für Anästhesiologie und Intensivmedizin
Fleischmannstraße 42-44
D-17487 Greifswald

In dieser Studie untersuchen wir Ihre individuelle Angstreaktion unter Einfluss von Ohrakupunktur in Prüfungssituationen. Aus der Forschung und klinischen Praxis ist bekannt, dass Ohrakupunktur angstlindernde Eigenschaften besitzt, obwohl die Datenlage dazu widersprüchlich ist. Mit unserer Studie möchten wir die Größe des angstlindernden Effekts der Akupunktur am Außenohr untersuchen. Die standardisierten Angstempfindungen werden sowohl psychisch, mit dem sogenannten State-Trait-Angstinventar (STAI) und einer visuellen Angstskala (VAS), als auch physisch, durch die Messung der Herzfrequenz, des Blutdruckes sowie des Speichelcortisols erhoben. Die Durchführung der Akupunktur erfolgt am Tag vor der Prüfung mit Dauernadeln, welche bis nach dem Testat im Ohr verbleiben und nach Bedarf eigenständig stimuliert werden können.

Was ist STAI?

Das State-Trait-Angstinventar ist eine Methode zur Erhebung von Angst als einen emotionalen Zustand (State) und Angst als Eigenschaft (Trait). Die Zustandsangst ist gekennzeichnet durch Nervosität, Angespanntheit, Besorgtheit, innere Unruhe, Angst vor zukünftigen Ereignissen sowie durch eine erhöhte Aktivität des autonomen Nervensystems. Angst als Eigenschaft bezieht sich demgegenüber auf interindividuelle Differenzen in der Bewertung der Bedrohlichkeit einer Situation.

Wie wird der Test durchgeführt?

Das STAI setzt sich aus einem Fragebogen aus je 20 Fragen zu Zustandsangst und je 20 Fragen zu Angst als Eigenschaft zusammen.

Was ist VAS?

Die visuelle Angstskala dient der Erhebung von subjektiven Empfindungen. Es handelt sich bei der VAS um eine horizontale Linie welche durch extreme Endpunkte von 0 (überhaupt nicht ängstlich) bis 100 (vollkommen ängstlich) quantifiziert wird.

Wie wird der Test durchgeführt?

Ziel ist es, seinen subjektiv empfundenen Angstzustand auf dieser Skala von 0 (überhaupt nicht ängstlich) bis 100 (vollkommen ängstlich) durch Markierung mit einem Stift fest zu legen.

Was ist Speichelcortisol?

Im menschlichen Speichel befindet sich das Stressormon Cortsiol. Dieses Hormon ist Produkt der Nebennierenrinde und wird bei erhöhter Stressreaktion freigesetzt. Es unterliegt einem zirkadianem Rhytmus.

Wie wird der Test durchgeführt?

Am Morgen des Testates vor dem Frühstück und vor dem Zähneputzen wird durch Beißen auf das Wattestäbchen der Salivette Speichel aus dem Mundraum gewonnen. Dabei bitte darauf achten, dass bei Entnahme und Verschließen die Spitze des Wattestäbchens die Verpackung nicht berührt.

Was wird mit den Werten gemacht?

Die Daten werden im Rahmen einer Doktorarbeit ausgewertet und können Ihnen nicht direkt im Anschluss an den Test mitgeteilt werden. Sind Sie an den ausgewerteten Daten interessiert, können wir Ihnen diese per Email zuschicken.

Wie lange wird der Test/Versuch dauern?

Der gesamte Versuch wird drei mal zwei Tage umfassen. Am Tag vor dem Testat werden wir Ihnen an unserem Standpunkt in der Unibibliothek die Ohrakupunkturnadeln einsetzen. Die gesamte Prozedur mit der Erfassung der physiologischen und psychologischen Parameter wird in etwa eine halbe Stunde in Anspruch nehmen. Am Tag des Testates bitten wir Sie, am Morgen den Speichelcortisoltest durchzuführen und ein weiteres Mal die Fragebögen auszufüllen, welches Sie bequem zu Hause erledigen können. Des Weiteren werden wir Ihren Blutdruck und Ihre Herzfrequenz etwa eine halbe Stunde vor Ihrem jeweiligen Testat erfassen.

Was ist Ohrakupunktur?

Die Ohrakupunktur ist eine der Reflextherapien, die über periphere sensorische Stimulation der Ohrmuschelareale mit Dauernadeln zu den Wirkungen in entfernten Körperregionen führt. Unerwünschte Wirkungen (Nebenwirkungen) der Akupunktur können sein: Schmerzen beim Einstich oder Entfernen der Nadel, kleine Blutungen, sehr selten (0,001%): Schwindel, Blutdruckabfall, Bradykardie, Tachykardie, psychische Reaktionen, lokal: Chondritis.

Untersuchungsablauf

Tag 1: Wir zeigen Ihnen den Untersuchungsraum sowie die Geräte zum Durchführen unserer Untersuchungen. Beginnen werden wir mit dem Ausfüllen der Fragebögen. Dem schließt sich die Messung der physiologischen Parameter an. Nun erfolgt die Ohrakupunktur, welche durch einen erfahrenen Akupunkteur durchgeführt wird. Abschließend werden noch einmal Herzfrequenz und Blutdruck gemessen. Zu Hause füllen Sie die Fragebögen erneut aus.

Tag 2: Wir bitten Sie, am Morgen vor dem Testat mit Hilfe des Wattestäbchens Speichel aus dem Mundraum zu entnehmen und die Fragebögen ein drittes und letztes Mal auszufüllen. Bitte bringen Sie die ausgefüllten Fragebögen und den Probenträger mit zu Ihrem Testat in die Anatomie, wo wir sie einsammeln werden. Des Weiteren werden wir ca. 30 min vor Ihrem Testat Ihren Blutdruck und die Herzfrequenz messen.

Studienprotokoll (Ohrakupunktur zur Linderung von Prüfungsangst)

Nr: Name: Datum:

Nr	Parameter	Vor Akupunktur Tag1	Nach Akupunktur Tag1	Nach Akupunktur Tag2	Letzte Nacht in h	Letzte Nacht Einschlafprobleme	Letzte Nacht Durchsschlafprobleme	Letzte Nacht Schlafqualität	Letzte Woche Schlaf in h	Letzte Woche Einschlafprobleme	Letzte Woche Durchschlafprobleme	Letzte 6 Monate Schlaf in h	Letzte 6 Monate Einschlafprobleme	Letzte 6 Monate Durchschlafprobleme	Bemerkungen
1	HF				xxxxxxxx<br>xxxxxxxx	xxxxxxxx<br>xxxxxxxx	xxxxxxxx<br>xxxxxxxx	xxxxxxxx<br>xxxxxxxx	xxxxxxxx<br>xxxxxxxx	xxxxxxxx<br>xxxxxxxx	xxxxxxxx<br>xxxxxxxx	xxxxxxxx<br>xxxxxxxx	xxxxxxxx<br>xxxxxxxx	xxxxxxxx<br>xxxxxxxx	
2	RR				xxxxxxxx<br>xxxxxxxx	xxxxxxxx<br>xxxxxxxx	xxxxxxxx<br>xxxxxxxx	xxxxxxxx<br>xxxxxxxx	xxxxxxxx<br>xxxxxxxx	xxxxxxxx<br>xxxxxxxx	xxxxxxxx<br>xxxxxxxx	xxxxxxxx<br>xxxxxxxx	xxxxxxxx<br>xxxxxxxx	xxxxxxxx<br>xxxxxxxx	
3	STAI X1				xxxxxxxx	xxxxxxxx	xxxxxxxx	xxxxxxxx	xxxxxxxx	xxxxxxxx	xxxxxxxx	xxxxxxxx	xxxxxxxx	xxxxxxxx	
4	STAI X2		xxxxxxx	xxxxxxx	xxxxxxxx	xxxxxxxx	xxxxxxxx	xxxxxxxx	xxxxxxxx	xxxxxxxx	xxxxxxxx	xxxxxxxx	xxxxxxxx	xxxxxxxx	
5	VAS				xxxxxxxx	xxxxxxxx	xxxxxxxx	xxxxxxxx	xxxxxxxx	xxxxxxxx	xxxxxxxx	xxxxxxxx	xxxxxxxx	xxxxxxxx	
6	Cortisol	xxxxxxx	xxxxxxxx		xxxxxxxx	xxxxxxxx	xxxxxxxx	xxxxxxxx	xxxxxxxx	xxxxxxxx	xxxxxxxx	xxxxxxxx	xxxxxxxx	xxxxxxxx	
7	Schlaf	xxxxxxx	xxxxxxx	xxxxxxx											
8	Testergebnis	xxxxxxx	xxxxxxx	xxxxxxx	xxxxxxx	xxxxxxx	xxxxxxx	xxxxxxx	xxxxxxx	xxxxxxx	xxxxxxx	xxxxxxx	xxxxxxx	xxxxxxx	
9	Akupunktur: Verum/Placebo	xxxxxxx<br>xxxxxxx	xxxxxxxx<br>xxxxxxxx	xxxxxxxx<br>xxxxxxxx	xxxxxxxx<br>xxxxxxxx	xxxxxxxx<br>xxxxxxxx	xxxxxxxx<br>xxxxxxxx	xxxxxxxx<br>xxxxxxxx	xxxxxxxx<br>xxxxxxxx	xxxxxxxx<br>xxxxxxxx	xxxxxxxx<br>xxxxxxxx	xxxxxxxx<br>xxxxxxxx	xxxxxxxx<br>xxxxxxxx	xxxxxxxx<br>xxxxxxxx	

Untersucher: Hiermit bestätige ich, dass die Daten in das Protokoll korrekt eingetragen sind.

Erklärung:

Untersucher 1:

Nadeln entfernt am…………um………Uhr durch…………………… (Unterschrift)
