# Supplementary material for: Auricular Acupuncture for Exam Anxiety in Medical Students—A Randomized Crossover Investigation
Source: PLoS One. 2016 Dec 29;11(12):e0168338. doi: 10.1371/journal.pone.0168338 (PMC5198977; doi:10.1371/journal.pone.0168338)
Supplement: S2 File — (PDF) [file pone.0168338.s003.pdf]

Downloaded from http://aim.bmj.com/ on April 5, 2016 - Published by group.bmj.com

Auricular acupuncture for pre-exam anxiety in medical students: a prospective observational pilot investigation

Catharina Klausenitz, Thomas Hesse, Henriette Hacker, Klaus Hahnenkamp, Taras Usichenko

Department of Anaesthesiology, Intensive Care Medicine, Emergency Medicine and Pain Medicine, University Medicine of Greifswald, Greifswald, Germany

Correspondence to Dr Taras Usichenko, Department of Anaesthesiology, Intensive Care Medicine, Emergency Medicine and Pain Medicine, University Medicine of Greifswald, Ferdinand-Sauerbruch-Strasse, Greifswald D-17475, Germany; taras@uni-greifswald.de

Accepted 15 September 2015
Published Online First 5 October 2015

To cite: Klausenitz C, Hesse T, Hacker H, *et al*. *Acupunct Med* 2016;**34**:90–94.

ABSTRACT

Objective Auricular acupuncture (AA) is effective for the treatment of preoperative anxiety. We aimed to study the feasibility and effects of AA on exam anxiety in a prospective observational pilot study.

Methods Healthy medical students received bilateral AA using indwelling fixed needles at points MA-IC1, MA-TF1, MA-SC, MA-AH7, and MA-T on the day before an anatomy exam. The needles were removed after the exam. Anxiety levels were measured using the State-Trait-Anxiety Inventory (STAI) and a 100 mm visual analogue scale (VAS-100) before and after the AA intervention and once again immediately before the exam. The duration of sleep on the night before the exam was recorded and compared to that over the preceding 1 week and 6 months (all through students' recollection). In addition, blood pressure, heart rate and the acceptability of AA to the students were recorded.

Results Ten students (all female) were included in the final analysis. All tolerated the needles well and stated they would wish to receive AA again for exam anxiety in the future. Exam anxiety measured using both STAI and VAS-100 decreased by almost 20% after AA.

Conclusions AA was well accepted, the outcome measurement was feasible, and the results have facilitated the calculation of the sample size for a subsequent randomised controlled trial.

INTRODUCTION

Exam (or test) anxiety is a form of situational anxiety that is a common problem among university students.[1 2] Exam anxiety often causes undesirable somatic and mental symptoms and may negatively influence academic results.[2–4] Various cognitive, mindfulness-based, behavioural and affect-regulating interventions, as well as electroacupuncture, have been demonstrated to be effective in reducing exam anxiety and stress among university students,[4–6] and it has been suggested that these improve academic performance.[7] However, these methods are time-consuming, which precludes the widespread use of cognitive and behavioural interventions in the treatment of anxiety immediately before an upcoming exam.[4–6]

Auricular acupuncture (AA) is a complementary medicine technique that is physiologically based on the stimulation of cranial nerves.[8] It has already been successfully used to treat situational anxiety in clinical settings.[9–11] Moreover, Ogal *et al*[12] used AA to relieve exam anxiety in medical students in a prospective observational study without a control group. However, the self-created questionnaire that was used to evaluate anxiety and stress in this investigation (within 30 min of AA only) was not previously validated. Thus the data from Ogal *et al* cannot be used to calculate the sample size for a controlled investigation.

Therefore, we conducted a pilot investigation of AA for the treatment of exam anxiety to test its acceptability, the feasibility of outcome measurements, and to provide the necessary data to calculate the sample size for a subsequent randomised controlled trial (RCT).

PARTICIPANTS AND METHODS

Design and participants

This prospective observational pilot investigation was performed at the Department of Anaesthesiology of the University

Downloaded from http://aim.bmj.com/ on April 5, 2016 - Published by group.bmj.com

Medicine Greifswald, Germany, in January 2012. The participants were recruited via an announcement according to the following eligibility criteria: acupuncture naïve medical students undergoing an oral anatomy exam, without any history of alcohol abuse or use of opioid or psychotropic medication, with an American Society of Anesthesiologists physical status score of I–II. None of the students was taking any medications or recreational drugs at the time of the study. The protocol of the study was approved by the Institutional Ethics Committee of the University Medicine Greifswald (reference no. BB 069/15). Participants who agreed to participate, and gave informed (written) consent, received AA with indwelling fixed needles in the evening of the day before the anatomy exam. The AA needles remained in situ until the next day and were removed immediately after the exam (figure 1).

Auricular acupuncture

Licensed acupuncturists with more than 5 years' experience with the technique applied AA bilaterally at the acupuncture points MA-IC1 (Lung), MA-TF1 (ear Shenmen), MA-SC (Kidney), MA-AT1 (Subcortex), and MA-TG (Adrenal gland) (figure 2 and table 1) according to the AA nomenclature of the WHO[13] and French classification,[14] respectively. The acupuncture points were chosen based on data from previous investigations using AA to treat preoperative and exam anxiety[9–12] and on neurophysiological considerations.[8] Indwelling fixed 'New Pyonex' needles (length 1.5 mm, diameter 0.22 mm; Seirin Corp, Shizuoka City, Japan) were used for AA. The participants were instructed by the acupuncturist to stimulate the auricular needles for 3–5 min if they felt anxious.

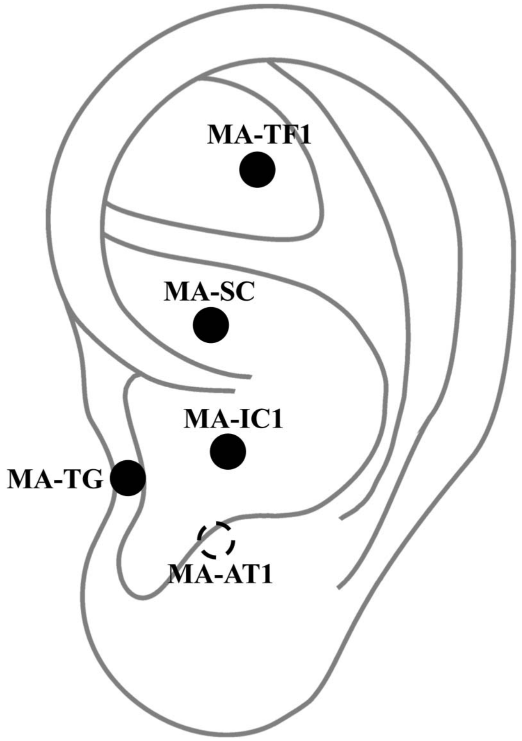

Figure 2 Auricular acupuncture (AA) points used for the treatment of exam anxiety in medical students. The following AA points were chosen based on previous reports[9–12]: MA-IC1 (Lung), MA-TF1 (ear Shenmen), MA-SC (Kidney), MA-AT1 (Subcortex), and MA-TG (Adrenal gland). Further information about point location is provided in table 1.

Data analysis

Exam anxiety was measured: (1) before AA; (2) in the evening of the day before the exam with the needles in situ; (3) and immediately before the anatomy exam the following day (figure 1) using the German version of the State-Trait Anxiety Inventory (STAI[15]), ranging from 20 (=no anxiety) to 80 (=maximum imaginable anxiety), and a 100 mm visual analogue scale (VAS-100), where 0=no anxiety and 100=maximum imaginable anxiety. Blood pressure and heart rate were measured before and after AA. After completion of the exam, students were also asked to recall their duration and quality of sleep the night before as well as 6 months and 1 week before the exam, and to rate the acceptability of AA for the treatment of exam anxiety (based on whether they would wish to receive the treatment again in the future). Statistical analysis was performed using SPSS-Statistics Software for Mac (V.19.0). Pre- and post-AA measurements were compared with Student's t test for paired samples. All data are presented as mean±SD unless otherwise stated.

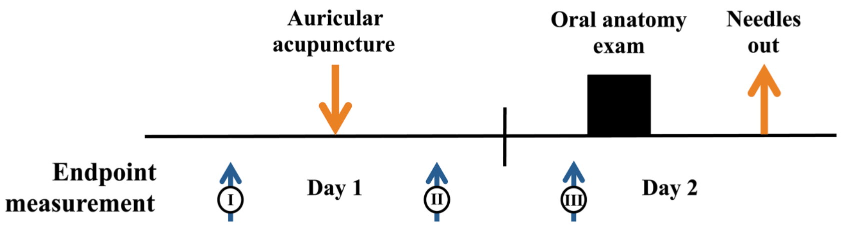

Figure 1 Timeline of the pilot investigation including endpoint measurements. Auricular acupuncture was performed in the evening before the day of exam using indwelling fixed needles, which remained in situ and were removed immediately after the exam. Exam anxiety was measured using the State-Trait-Anxiety Inventory (STAI) and 100 mm visual analogue scale (VAS-100) at time points I, II and III, as was heart rate and blood pressure. Duration and quality of sleep (over the course of the preceding 1 night, 1 week and 6 months) were enquired about at time point III.

Downloaded from http://aim.bmj.com/ on April 5, 2016 - Published by group.bmj.com

Table 1 Description of auricular acupuncture points used in the study

Nomenclature: WHO[13]	Nomenclature: French[14]	Location	Classical indications
MA-IC1	Lung	Around the central depression of the inferior concha	Respiratory diseases, withdrawal syndrome
MA-TF1	Shenmen	In the triangular fossa, at the superior aspect of the bifurcating point between the superior and inferior antihelix crura	Insomnia, acute and chronic pain syndromes, withdrawal syndrome, urticaria, asthma
MA-SC	Kidney	In the superior concha, inferior to the bifurcating point between the superior and inferior antihelix crura	Lumbago, tinnitus, asthma
MA-AT1	Subcortex	On the medial aspect of the antitragus	Pain, inflammation, psychiatric disorders
MA-TG	Adrenal gland	At the tip of the lower protuberance on the border of the tragus	Hypotension, parotitis, allergic diseases

RESULTS

A total of 15 students were invited to participate, four of whom declined and 11 of whom were included (figure 3). All 11 volunteers happened to be female. One student receiving AA missed the anatomy exam because of acute gastroenteritis and was therefore excluded. Therefore, data from a total of 10 female participants aged 23±3 years were available for analysis. All students tolerated AA well and stated that they would wish to receive the treatment again for exam anxiety in the future.

Exam anxiety, measured using STAI and VAS-100, decreased after AA relative to baseline. STAI scores of state anxiety decreased from 58±10 to 48±11 ($p=0.006$). Anxiety values rated on the VAS-100 reduced from 59±22 to 49±20 ($p=0.04$; table 2), although anxiety test results on the day of the exam did not differ significantly compared to before AA treatment. Trait anxiety measured using STAI did not change during the course of the investigation and averaged 40±9 across all three measurements. Systolic blood pressure decreased from 130±12 mm Hg before AA to 121±11 mm Hg after AA ($p=0.02$). Neither diastolic blood pressure nor heart rate changed significantly over the course of the investigation. Students reported fewer hours of sleep during the week before the exam compared to the preceding 6 months; however, the incidence of other sleep problems remained unchanged.

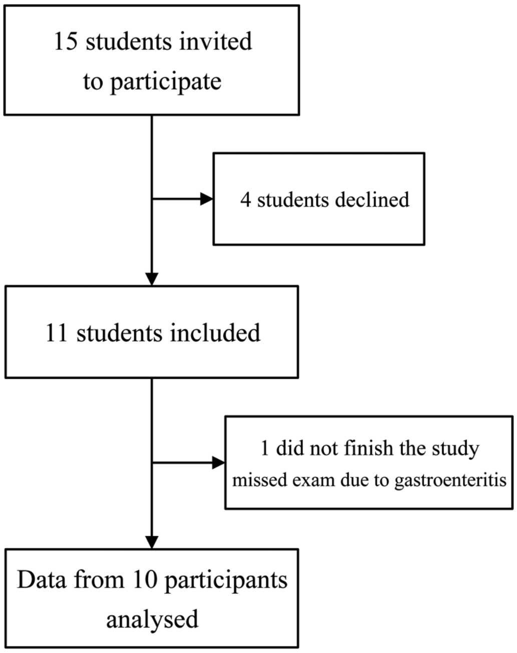

Figure 3 Flow diagram of the study.

DISCUSSION

In this observational pilot study, AA (applied using indwelling fixed needles) was well accepted by the medical students for the treatment of exam anxiety. The treatment protocol, derived from evidence-based prescriptions of AA for treatment of anxiety, was easy to perform before the exam and appeared safe in terms of potential side effects and complications, even with overnight retention of the needles.

Exam anxiety, measured using STAI and VAS-100, decreased by almost 20% after AA. The effect size observed in the present study is comparable to previous investigations in which AA has been used to treat preoperative or dental anxiety.[9–11] For example, Karst *et al*[10] reported that the STAI state anxiety score decreased from 51±9 at baseline to 42±13 after AA treatment of dental anxiety in 19 patients. Likewise, Michalek-Sauberer *et al*[11] demonstrated a reduction in STAI state anxiety from 55±11 at baseline to 47 ±10 after AA in 61 patients scheduled for dental procedures.

Notably, on the day of the exam, state anxiety scores increased back to almost the same level as that observed before AA treatment the previous day. Since anxiety increases constantly as the exam approaches,[16] one would have expected state anxiety in our study to do the same. However, the absence of an appropriate control group prevents us from drawing definitive conclusions. In order to differentiate the natural course of exam anxiety from the effect of AA, the inclusion of an untreated control group is necessary. Consequently, in our subsequent RCT we plan to use a crossover design testing three different conditions: (1) verum AA; (2) placebo AA; and (3) no treatment. According to the teaching schedule, each medical student volunteer will undergo three comparable

Downloaded from http://aim.bmj.com/ on April 5, 2016 - Published by group.bmj.com

Table 2 Endpoints of the study (n=10)

Endpoints	Day 1 Before AA	Day 1 After AA	Day 2 before exam
State anxiety (STAI)	***58±10***	***48±11*** **	51±13
Trait anxiety (STAI)	41±10	40±9	39±8
Anxiety (VAS-100)	***59±22***	***49±20****	50±27
Heart rate (bpm)	78±14	72±10	
Systolic blood pressure (mm Hg)	***130±12***	***121±11****	
Diastolic blood pressure (mm Hg)	77±9	73±10	
	**Preceding 6 months**	**Preceding week**	**Night before exam**
Length of night sleep (h) by student's own recollection	***7.9±0.8***	***6.9±1.7****	7.3±2.3
Problems falling asleep	4	6	5
Awakening during the night	4	6	5

All data are presented as mean±SD or number of participants (n=10 in total). Bold italic font indicates statistically significant differences relative to baseline (before AA); *p<0.05; **p<0.01.
AA, auricular acupuncture; bpm, beats/min; STAI, State-Trait Anxiety Inventory; VAS, visual analogue scale.

anatomy exams at intervals of 1 month, each under one of the three aforementioned conditions (1, 2 or 3) in a randomised order. The intra-individual comparison of outcome measures that can be made with the crossover design will control for variation between participants in terms of individual characteristics, thereby allowing for a smaller sample size. State anxiety (measured using STAI) was feasible as the main outcome measure in this investigation and will be used for the estimation of the sample size for our RCT.

As expected, STAI trait anxiety scores did not change during the course of the investigation. This is unsurprising as they are thought to reflect a stable personality characteristic that remains constant over time and between events.[15] The mean value of trait anxiety in the study group was 40±9, which is slightly higher than the mean in the general female population aged between 15 and 29 years (36±10), but below the critical cut-off of 48±10 for the diagnosis of specific phobia disorder.[15]

The fact that only females volunteered to participate in our study might be due to the fact that female medical students consistently reveal decreased self-confidence and increased anxiety.[17] Unfortunately the absence of any male subjects limits the ability to extrapolate the results to a mixed population, and it will be important to include students of both sexes in our subsequent RCT to avoid gender bias.

The observational longitudinal design and small size are the main limitations of the present investigation, although it should be remembered that this study was designed with the specific purpose to test the methodology and yield pilot data to support further randomised controlled investigations. Another potential limitation is the lack of measurement of stress-specific biomarkers (such as cortisol, catecholamines and salivary α-amylase) or continuous monitoring of heart rate variability and electrodermal activity. Future studies should consider including these potentially more objective outcome parameters in addition to stress/anxiety questionnaires.

In conclusion, AA was well accepted by medical student volunteers as a method of treating exam anxiety and the outcome measurements chosen were feasible. The results have facilitated the calculation of the sample size for a subsequent RCT.

Acknowledgements The authors would like to thank the students who participated in this study.

Contributors All authors contributed to the writing and redrafting of the manuscript. CK had the original idea. TU prepared the study. TH and TU conducted the study. CK, HH, KH and TU analysed the results.

Competing interests None declared.

Patient consent Obtained.

Ethics approval This study was approved by the Institutional Ethics Committee of the University Medicine Greifswald (reference number BB 069/15) and was carried out in accordance with the principles of the Declaration of Helsinki.

Provenance and peer review Not commissioned; externally peer reviewed.

REFERENCES

1 Eisenberg D, Hunt J, Speer N. Mental health in American colleges and universities: variation across student subgroups and across campuses. *J Nerv Ment Dis* 2013;201:60–7.
2 Latas M, Pantic M, Obradovic D. Analysis of test-anxiety in medical students. *Med Pregl* 2010;63:863–6.
3 Zhang Z, Su H, Peng Q, *et al*. Exam anxiety induces significant blood pressure and heart rate increase in college students. *Clin Exp Hypertens* 2011;33:281–6.
4 Dias M, Vellarde GC, Olej B, *et al*. Effects of electroacupuncture on stress-related symptoms in medical students: a randomised placebo-controlled study. *Acupunct Med* 2014;32:4–11.
5 Neuderth S, Jabs B, Schmidtke A. Strategies for reducing test anxiety and optimizing exam preparation in German university students: a prevention-oriented pilot project of the University of Würzburg. *J Neural Transm* 2009;116:785–90.

Downloaded from http://aim.bmj.com/ on April 5, 2016 - Published by group.bmj.com

6 Regehr C, Glancy D, Pitts A. Interventions to reduce stress in university students: a review and meta-analysis. *J Affect Disord* 2013;148:1–11.

7 Ramirez G, Beilock SL. Writing about testing worries boosts exam performance in the classroom. *Science* 2011;331:211–3.

8 Usichenko TI, Mustea A, Pavlovic D. On ears and Head. *Acupunct Med* 2010;28:165–6.

9 Wang SM, Peloquin C, Kain ZN. The use of auricular acupuncture to reduce preoperative anxiety. *Anesth Analg* 2001;93:1178–80.

10 Karst M, Winterhalter M, Münte S, *et al*. Auricular acupuncture for dental anxiety: a randomized controlled trial. *Anesth Analg* 2007;104:295–300.

11 Michalek-Sauberer A, Gusenleitner E, Gleiss A, *et al*. Auricular acupuncture effectively reduces state anxiety before dental treatment—a randomised controlled trial. *Clin Oral Investig* 2012;16:1517–22.

12 Ogal H, Ogal M, Hafer J, *et al*. Beginn der Anxiolyse und Relaxation unter Ohrakupunktur. *Deutsche Zeitschrift für Akupunktur* 2004;47:6–12.

13 World Health Organization. *Third WHO Regional Working Group on Standardization of Acupuncture Nomenclature*. Seoul, 1987.

14 Nogier PFM. *Traité d'auriculotherapie*. Moulinlés-Metz: Maisonneuve, 1972

15 Spielberger CD. *Manual for state-trait anxiety inventory (STAI: Form Y)*. Palo Alto, California: Consulting Psychologist Press, 1983.

16 Brockmeyer H. *Prüfungsangst: eine experimentelle Studie zur Wirkung der Ohrakupuntur auf Psyche und Hormone*. Essen: KVC Verl., 2005:79.

17 Blanch DC, Hall JA, Roter DL, *et al*. Medical student gender and issues of confidence. *Patient Educ Couns* 2008;72:374–81.

Downloaded from http://aim.bmj.com/ on April 5, 2016 - Published by group.bmj.com

Auricular acupuncture for pre-exam anxiety in medical students: a prospective observational pilot investigation

Catharina Klausenitz, Thomas Hesse, Henriette Hacker, Klaus Hahnenkamp and Taras Usichenko

Acupunct Med 2016 34: 90-94 originally published online October 5, 2015
doi: 10.1136/acupmed-2015-010887

Updated information and services can be found at:
http://aim.bmj.com/content/34/2/90

These include:

References
This article cites 13 articles, 3 of which you can access for free at:
http://aim.bmj.com/content/34/2/90#BIBL

Email alerting service
Receive free email alerts when new articles cite this article. Sign up in the box at the top right corner of the online article.

Notes

To request permissions go to:
http://group.bmj.com/group/rights-licensing/permissions

To order reprints go to:
http://journals.bmj.com/cgi/reprintform

To subscribe to BMJ go to:
http://group.bmj.com/subscribe/
